# Planarians as models to investigate the bioactivity of gold(I) complexes *in vivo*

**DOI:** 10.1038/s41598-018-34558-6

**Published:** 2018-11-01

**Authors:** Luiza G. Tunes, John M. Allen, Ricardo M. Zayas, Rubens L. do Monte-Neto

**Affiliations:** 1 Instituto René Rachou – Fiocruz Minas, Belo Horizonte, Minas Gerais, Brazil; 2 Department of Biology, San Diego State University, San Diego, California, USA

## Abstract

Gold(I)-containing complexes are used in drug discovery research for rheumatoid arthritis, cancer, and parasitic infections. In this study, we tested the bioactivity of gold(I) complexes *in vivo* using planarians. The planarian *Schmidtea mediterranea* possesses orthologues of tumor suppressor genes, such as *p53*, that, when silenced, cause deregulation of cell proliferation and apoptosis. In this context, we tested two triethylphosphine-gold(I) complexes (AdO and AdT) to determine if they can attenuate phenotypes that result from *p53* inhibition. First, we identified the drug concentration that did not affect survival or regeneration and evaluated the drug’s effect on cell division and apoptosis. We found that AdT treatment decreased the number of mitotic cells and that all drug treatments increased the number of apoptotic cells. We then performed *p53(RNAi)* and drug treatments concomitantly and observed the phenotype progression. Drug treatment increased survival three-fold and decreased apoptosis, which resulted in an attenuated phenotype. Our results indicate that planarians can be treated with gold(I) complexes, and that this treatment can diminish the *p53(RNAi)* phenotype and extend survival. In this work we show that planarians can be used as a model to study the *in vivo* effect of gold(I) complexes and to further investigate their mechanisms of action.

## Introduction

The use of gold for medicinal purposes dates back thousands of years, but with little scientific support until the 1960s when it was demonstrated that gold-containing compounds were valuable for the treatment of rheumatoid arthritis^1,2^. Subsequent studies uncovered that Auranofin, a gold(I)-containing drug with anti-inflammatory properties initially developed to treat chronic rheumatoid arthritis, has additional anti-cancer and anti-parasitic activities^3^. Several studies reported on the *in vivo* anti-cancer activity of Auranofin^4–8^ and there are ongoing clinical trials using Auranofin to treat ovarian, fallopian tube, peritoneal, and lung cancers^9^. The renewed focus on Auranofin led to the development of new families of bioactive gold compounds and their *in vitro* and *in vivo* anti-cancer activity demonstrates how promising this class of compounds are for drug discovery^10–14^.

Several mechanisms of action were implicated in the bioactivity of gold complexes including, anti-inflammatory activity, inhibition of cysteine proteases, and disruption of oxidative phosphorylation pathways^15,16^. The primary intracellular targets for gold complexes are enzymes responsible for redox homeostasis, such as Thioredoxin Reductase (TR)^3^, which is involved in the regulation of cellular proliferation, viability, and apoptosis, and is an important target for anti-cancer drug development^17^. To characterize the biological activity of new gold complexes we can utilize planarians as an *in vivo* model system. Planarians are free-living flatworms that are commonly used in pharmacology^18^ and are also a suitable model for cancer research;^19–24^ for example, the anti-cancer agent, rapamycin, effectively attenuates RNAi-induced hyper-proliferation and outgrowths in planarians^25,26^. Thus, the experimental accessibility of these organisms can be exploited to screen anti-cancer drug bioactivities. However, studies testing anti-parasitic or anti-cancer drugs are currently scarce^21,27^.

Here, we designed experiments using the planarian *Schmidtea mediterranea* to test the bioactivity of recently developed gold(I) complexes^28^ and to examine the toxicity and potential mechanisms of action of these complexes in a whole-organismal context. We postulate that this model will act complementarily to mammalian models, contributing information that would be difficult to obtain from cell lines and where using mice could be prohibitively laborious or expensive. An inherent property of the remarkable regenerative abilities of planarians is the capacity to tightly control their cell cycle and maintain genomic stability. This capacity is dependent in part on conserved pathways that include homologs of human tumor suppressor and DNA repair genes. Silencing of these homologs using RNAi provides a model for evaluating drug effects in a whole-organismal context and to study specific tumor suppression pathways. Thus, we investigated the effect of gold(I) complexes and their precursor in worms treated with RNAi for the tumor suppressor *p53*, a gene associated with regulation of cell proliferation in planarians^29^.

The gold(I) complexes used in this study were synthesized from a precursor chloro(triethilphosphine)gold(I) that has a linear P-Au-Cl coordination based on Auranofin. The adamantane ligand bound to oxadiazole forms the complex triethylphosphin[5-adamantyl-1,3,4-oxadiazole-2-thiolate(κS)]gold(I) (AdO) and to thiazolidine forms the complex triethylphosphin[(methyl-1-adamantane)1,3-thiazolidine-2-thione(κS)]gold(I) (AdT). These compounds, including the precursor have been tested *in vitro* and were selectively cytotoxic against tumor-derived cell lines^28^. To examine the bioactivity of the compounds *in vivo*, we incubated planarians with the compounds and found that AdT treatment decreased the number of mitotic cells, and all compounds increased the number of apoptotic cells in wild type worms. Thus, we hypothesized that the complexes would suppress the effects of RNAi against known tumor suppressor genes and that this experimental model would provide an insight into *in vivo* drug activity. Our experiments revealed that *p53(RNAi)* planarians survive longer when treated with gold(I) compounds when compared to control worms and showed an attenuated *p53(RNAi)* phenotype. The increased survival is likely caused by a decrease in apoptosis in treated worms, indicating that the compounds target a protein involved in regulating apoptosis independent of P53. We conclude that planarians are a practical model system that can validate anti-cancer drug activity *in vivo* and to examine drug effects on specific pathways. The experimental ease by which drugs can be delivered to *Schmidtea mediterranea*, combined with the genomic resources available for this organism, will be useful in studying mechanisms that underlie the effect of gold-containing compounds, and will contribute to anti-cancer and anti-parasitic drug discovery efforts.

## Methods

### Planarian husbandry

A clonal, asexual strain of *Schmidtea mediterranea* (CIW4) was maintained at 20 °C in Montjuïc salts (1.6 mM NaCl, 1.0 mM CaCl_2_, 1.0 mM MgSO_4_, 0.1 mM MgCl_2_, 0.1 mM KCl and 1.2 mM NaHCO_3_ prepared in nanopure water)^30^. Animals ranged in length from 3–6 mm and were starved for at least one week prior to all experiments.

### Drug treatments

The tolerance of the worms to AdO, AdT and the precursor was tested in concentrations ranging from 0.1 to 20 μM. The worms were soaked in 1X Montjuïc salts containing the drugs dissolved in DMSO (not exceeding 1% at final concentration) and monitored over a period of 14 days. For all other drug treatments, the worms were exposed to 0.1 μM of the drugs by soaking.

### Immunohistochemistry

Fixation and immunostaining with anti-phospho-Histone H3 (pH3) (1:2000, Cell Signaling) was performed as previously described^31^. Stained cells were visualized with Cyanine dye – Cy3-Tyramide – following incubation with goat anti-rabbit-HRP secondary antibodies (1:2000). TUNEL staining was performed as described in Pellettieri *et al*.^32^. Animals treated with terminal transferase enzyme were incubated with anti-digoxigenin-POD at 1:500 and the signal was developed using Cy3-Tyramide.

### Whole-Mount *In Situ* Hybridization

Animals were euthanized in 5% N-Acetyl Cysteine solution prior to fixation in 4% formaldehyde. Antisense RNA probes were synthesized as previously described^33^. Briefly, DNA templates were PCR amplified from cDNA clones in pBluescript II SK( + ) (Stratagene), from an available collection^34^. Antisense riboprobes labeled with digoxigenin were synthesized at 37 °C. Probes were purified via ethanol precipitation and whole-mount *in situ* hybridization (WISH) was performed in an InsituPro VS liquid handling robot (Intavis). Samples were incubated with an anti-digoxigenin AP antibody (Roche,1:2000) and developed with NBT/BCIP^33^.

### Cloning

The *p53* gene (accession number AY068713) was directionally cloned into pPR-T4P with primers containing overhangs homologous to the vector, using ligation independent cloning^35^. The *p53* primer sequences used were: forward primer 5′-CATTACCATCCCGGCCCAGCAATATATTACTTCGGC-3′ and reverse primer 5′-CCAATTCTACCCGCTGGTGCATCGGTCTATAGGG-3′.

### RNA interference

Plasmid templates in pPR-T4P were transformed into the RNase-*free E*. *coli* line HT115 (DE3) for double-stranded RNA production by using IPTG inducible promoters^36^. Green fluorescent protein *(gfp)* - a sequence not present in planarians - dsRNA was used as a control in all experiments. Animals were fed a dsRNA bacterial pellet mixed with an approximate 3:1 ratio of calf liver:water paste containing red food dye. Three to five RNAi feedings were performed over a period of two to five weeks, phenotypes were observed in homeostasis worms. All experiments were performed at least in duplicate.

### Image acquisition and processing

Fluorescent-labeled images were acquired with an Axiocam MRm camera mounted on a Zeiss Axio Observer.Z1 equipped with ApoTome or a Zeiss SteREO Lumar V.12. Live RNAi and *in situ* images were taken with a Leica DFC290 or DFC450 camera on a Leica M205 microscope. Images were only processed for brightness and contrast.

### Statistics

Quantification of pH3^+^ and TUNEL^+^ cells was done by manually counting cells on ImageJ software. Cell counts were normalized to the area of the worm. Student’s t-tests were performed, and graphs made on GraphPad Prism Version 6 (GraphPad Software). All graphs show the mean ± s.d.

## Results

### Planarians tolerate treatment with gold(I) complexes

**Figure 1:**
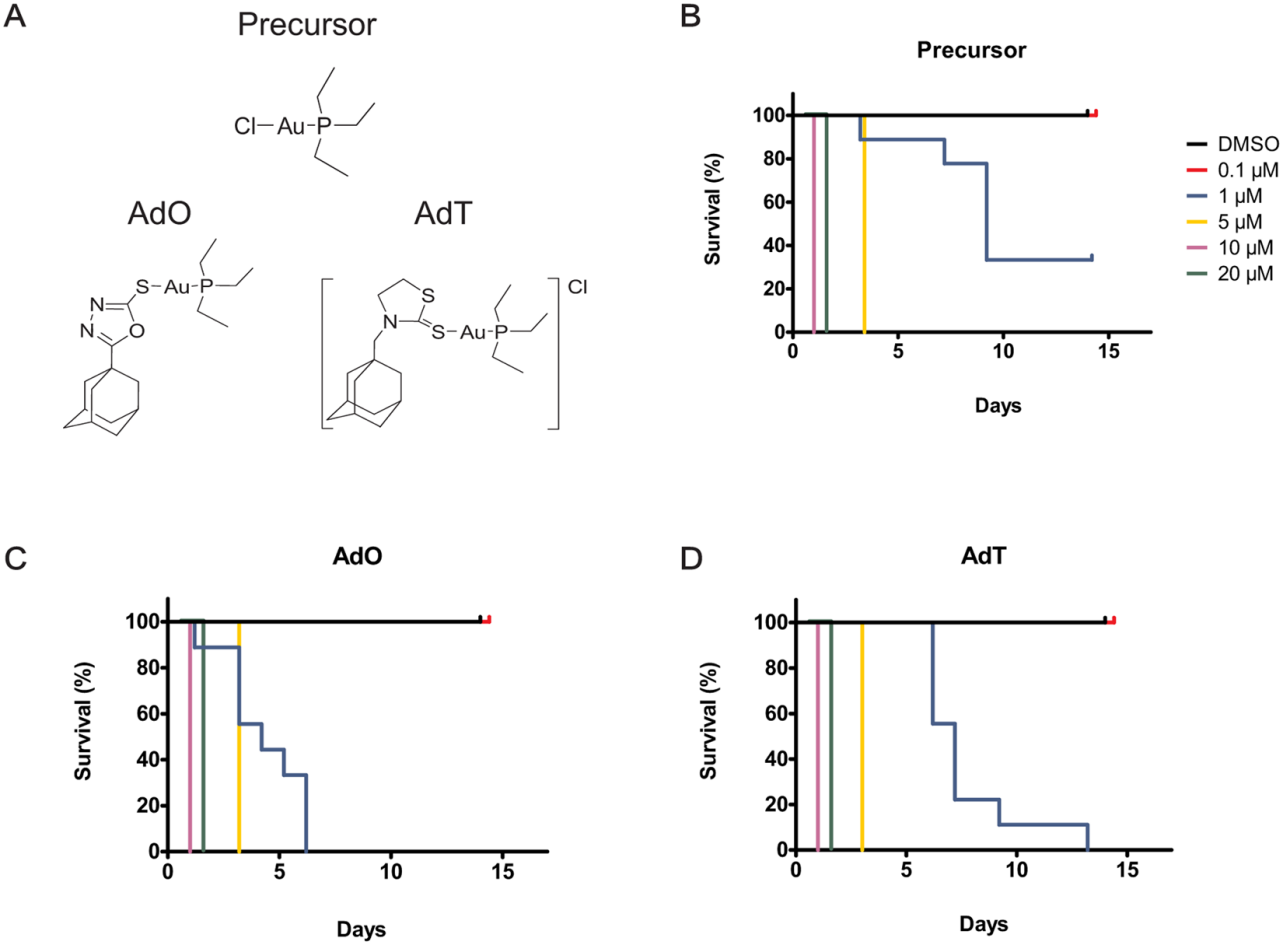
Planarians can tolerate treatment with gold(I) complexes. (**A**) Chemical structure of the precursor and the complexes AdO and AdT (adapted from Garcia *et al*.^28^). (**B–D**) Survival curves of worms treated for 14 days with varying concentrations of the precursor or the complexes AdO and AdT (n = 9 worms for each concentration and per treatment). The highest concentration of the complexes tolerated by the worms was 0.1 μM.

To study the effect of the gold(I) complexes (Fig. 1A) on planarians, we first determined the highest concentration of the drugs that the animals can tolerate. Asexual *Schmidtea mediterranea* were exposed to various concentrations of drugs for 14 days and monitored daily for health (worm integrity, photophobic behavior, locomotion) and survival. We found that AdO was more toxic than AdT or the precursor and that all drug concentrations above 0.1 μM were toxic and resulted in death of the worms by lysis (Fig. 1B–D). A dose of 0.1 μM was selected for all follow up experiments. To test if the complexes would interfere with regeneration, planarians were amputated anterior to the pharynx and allowed to regenerate in the presence of the compounds. All treatment groups regenerated normally, with formation of externally visible structures (blastema, eyespots and pigmentation) occurring at the same time-points after amputation as the vehicle DMSO control treatment groups.

**Figure 2:**
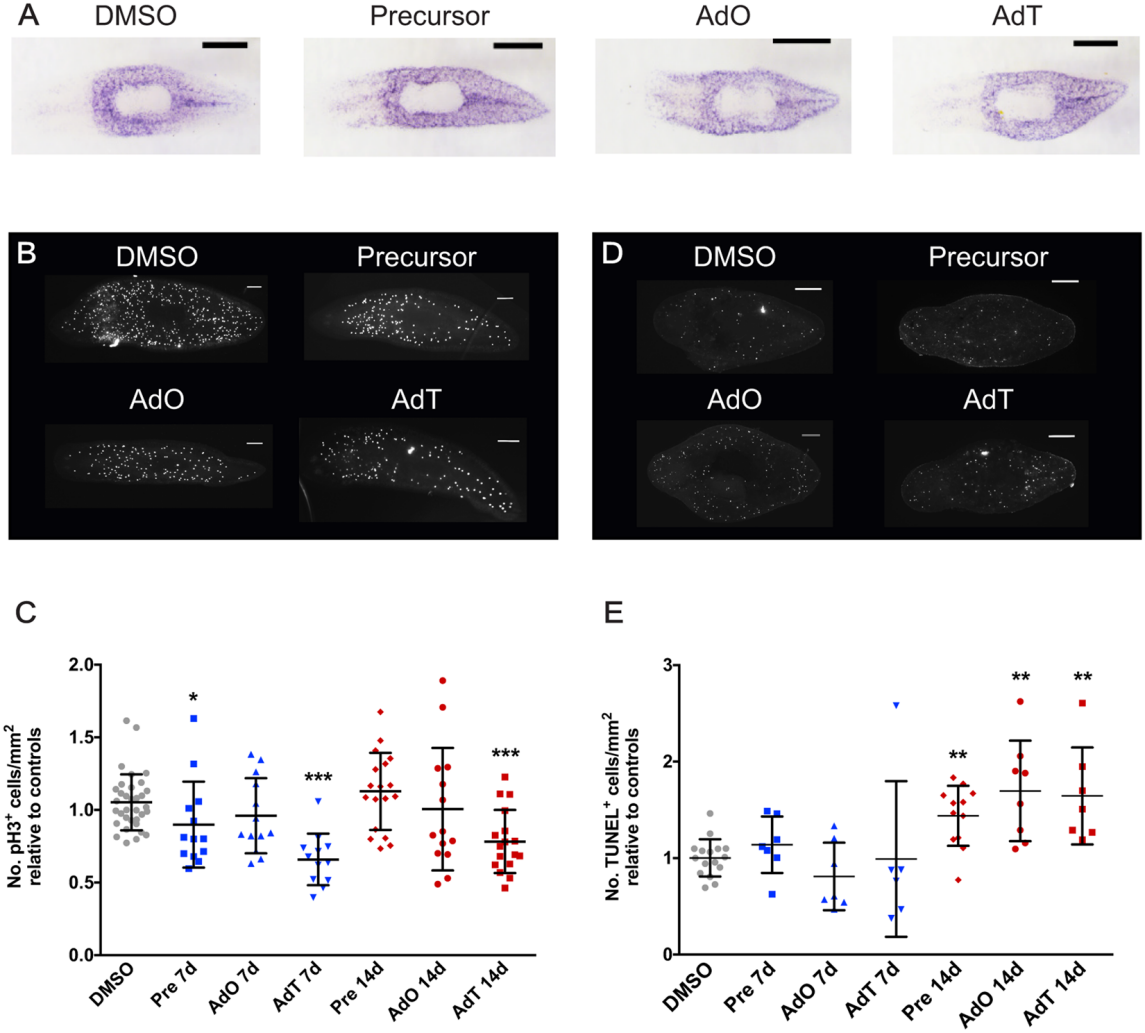
AdT reduces the number of mitotic cells and gold(I) complex treatment increases apoptosis. (**A**) WISH of stem cell-specific gene *piwi-1* showing that the stem cell population remains unaltered in worms treated with DMSO, precursor, AdO or AdT (n = 6 per treatment). (**B**) Worms treated 7 and 14 days with AdT have fewer pH3^+^ cells compared to DMSO treated worms. Representative images of pH3 staining of treated worms. (**C**) Quantification of the number of pH3^+^ cells, showing the reduced number of mitotic cells in worms treated with AdT (n ≥ 12 per time point, per treatment). (**D**) Representative images of TUNEL stained worms treated with DMSO, precursor, AdO, or AdT treated (n ≥ 7 per time point, per treatment). (**E**) Quantification of TUNEL^+^ cells of treated worms, showing increased apoptosis in planarians soaked with 0.1 μM of the drugs for 14 days. Scale bars = 200 µm.

To evaluate if the complexes would affect stem cells we examined expression of the stem cell-specific gene transcript *piwi-1*^37^ using WISH. We found that the drug treatments at a concentration of 0.1 μM had no obvious effect on *piwi-1* staining, which suggests that this concentration of drugs is not affecting the stem cell population (Fig. 2A). Because anti-cancer compounds can often cause a reduction in mitotic activity, we performed immunohistochemistry using the mitotic marker antibody, anti-pH3, to assess cell proliferation in worms treated with the gold(I) complexes. Worms exposed to 0.1 μM of the precursor or AdO showed no difference in mitotic rates, while AdT caused significant reduction in mitotic levels after 14 days of treatment (Fig. 2B, C). Furthermore, a common effect of anti-cancer drugs is an increase in apoptosis^38^. To investigate if our complexes affected cell survival, we performed TUNEL staining to mark DNA fragmentation as a direct evidence for apoptosis. Worms exposed to 0.1 μM of the drugs showed no difference in DNA fragmentation after 7 days of treatment. After 14 days, increased TUNEL staining was observed in all treated groups, indicating an upregulation of apoptosis (Fig. 2D, E).

These data show that the worms can tolerate treatment with 0.1 μM of the gold(I) complexes. This dose does not adversely affect stem cells and regeneration, but we found that apoptotic rates were increased with all three treatments and that proliferation was decreased after AdT treatment. This prompted us to investigate the potential of these complexes to counteract phenotypes that are observed after RNAi knockdown of genes that regulate the cell cycle and are implicated in cancer development in humans.

### Treatment with gold(I) complexes increases survival and decreases apoptosis in *p53-*deficient planarians

**Figure 3:**
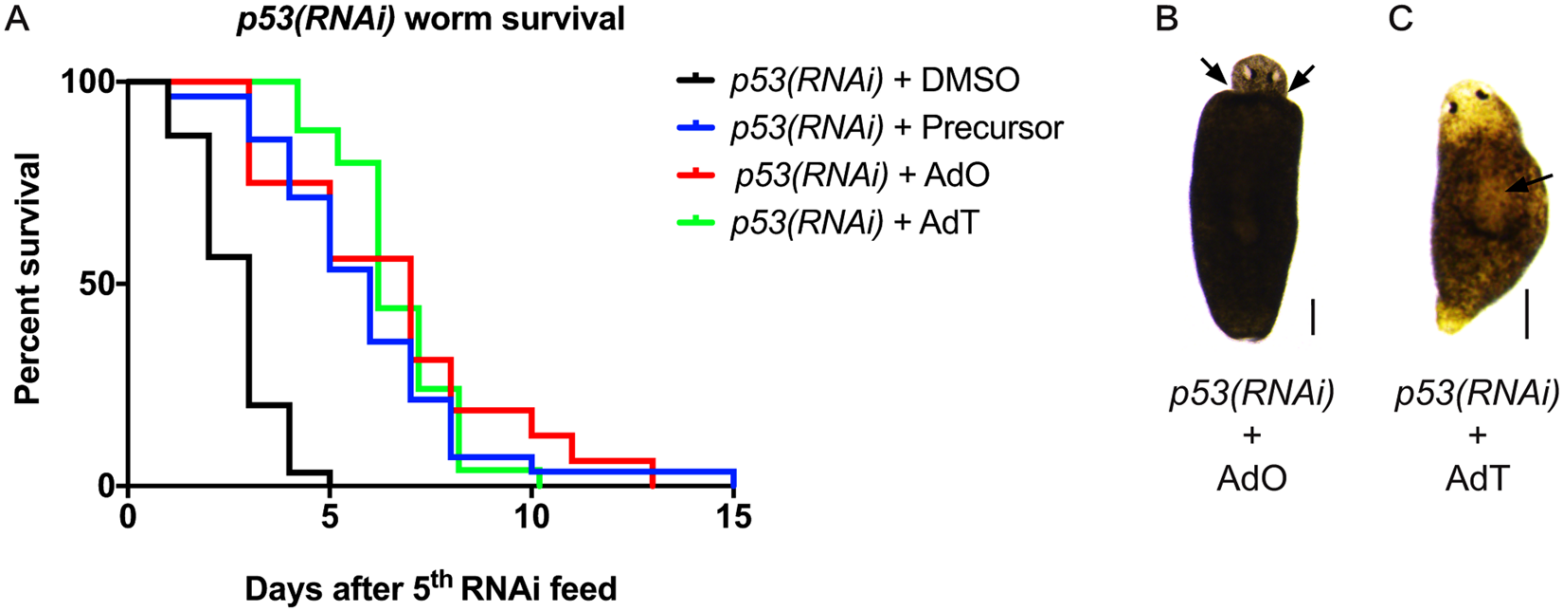
*p53(RNAi)* worms treated with gold(I)-based compounds have increased survival and an attenuated phenotype. (**A**) Survival curves of *p53(RNAi)* worms showing increased survival for worms treated with 0.1 μM of the drugs (n = 20 animals per treatment). Representative image of a worm treated with AdO showing head regression (indicated by arrows) (**B**) and a worm treated with AdT showing a dorsal outgrowth (marked by arrow) (**C**). Scale bars = 200 µm.

To study the effect of gold(I) complexes on planarian tumorigenesis-related phenotypes, we tested the drugs on *p53(RNAi)*-treated worms. We performed RNAi knockdowns and drug treatments concomitantly and observed the progression of the phenotypes, which are reported to be stem cell hyper-proliferation, tissue outgrowths and eventual death. We fully replicated the *p53(RNAi)* phenotype observed previously^24^ and *p53(RNAi)* worms treated with the compounds survived up to three times longer when compared to untreated ones (Fig. 3A). Moreover, while Pearson *et al*. (2010) observed head regression and outgrowths during regeneration of worms that were fed a lower dose of dsRNA (only 1 feed), we found that worms treated with the complexes required at least five feeds of RNAi before manifesting the same phenotypes (Fig. 3B, C). In addition to the increased survival, this indicates that the treatment attenuated the phenotype.

**Figure 4:**
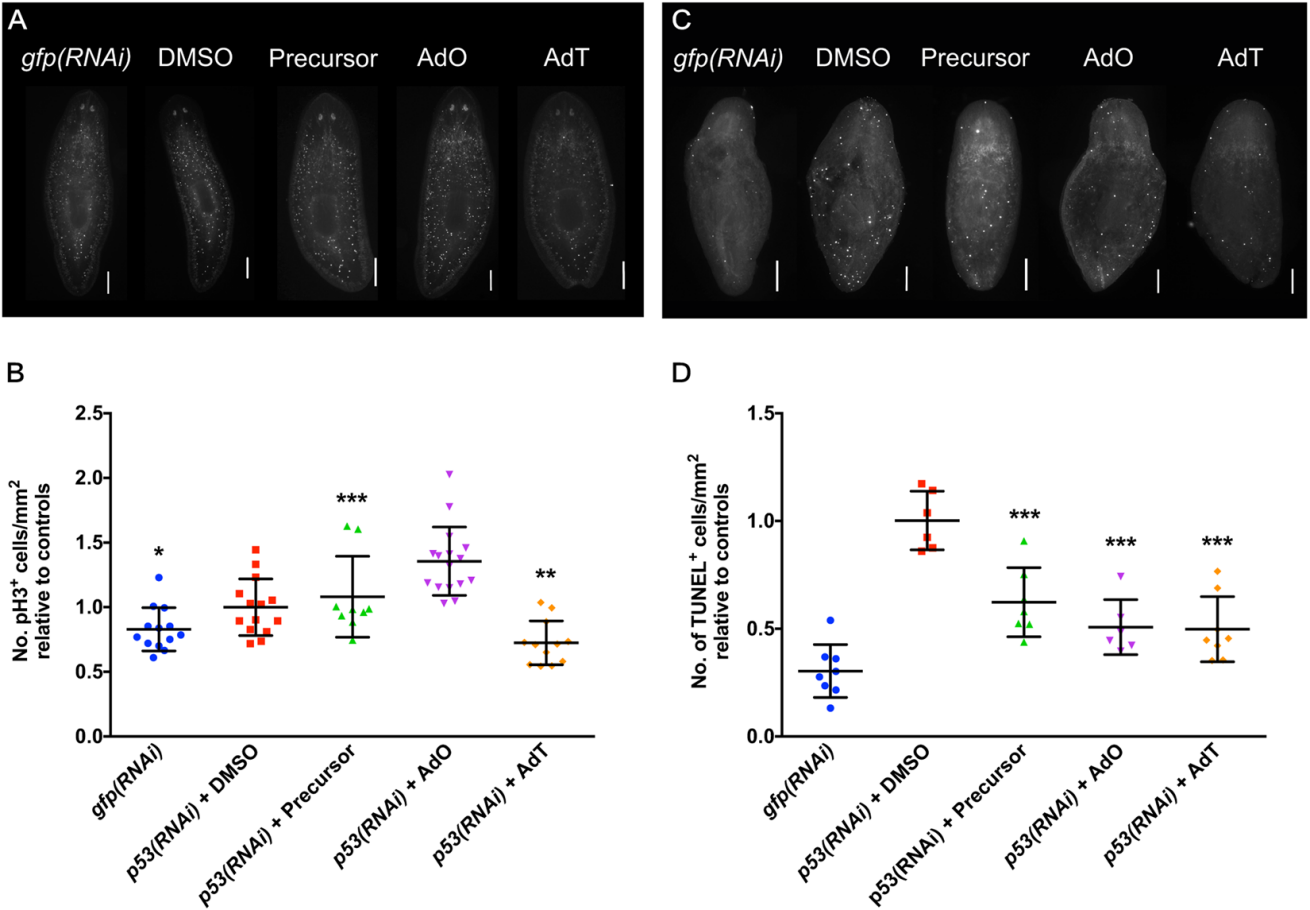
AdT treatment reduces the number of mitotic cells and treatment with gold(I) complexes reduces TUNEL staining in *p53(RNAi)* worms. (**A**) Representative images of pH3 staining of worms treated with *gfp*, DMSO, precursor, AdO and AdT (n ≥ 9 per treatment). (**B**) Graph summarizing quantification of pH3^+^ cells, showing the reduced number of mitotic cells in worms treated with 0.1 μM of AdT for 14 days when compared to *p53(RNAi)* worms treated with DMSO. (**C**) Representative images of TUNEL staining of worms treated with *gfp*, vehicle control, precursor, AdO and AdT (n ≥ 6 per treatment). (**D**) Graph summarizing quantification of TUNEL staining of treated worms, showing increased apoptosis in *p53(RNAi)* planarians and the decrease in apoptosis when worms were treated with 0.1 μM the drugs for 14 days. Scale bars in A = 200 µm.

To understand how the gold(I) complexes affect the cell cycle in *p53(RNAi)* worms and whether this can explain the attenuated phenotype upon treatment, we performed assays to quantify the number of mitotic and apoptotic cells. Similar to the results seen with the drug treatment alone (i.e. no RNAi knockdown), only AdT treatment decreased the mitotic rate in *p53(RNAi)* worms (Fig. 4A, B). The *p53(RNAi)* planarians had a 3-fold increase in apoptosis when compared to control *gfp(RNAi)*, which is expected, since the worms eventually die because of stem cell depletion. However, when treated with the precursor, AdO or AdT, the number of apoptotic cells was reduced by 37%, 49% and 53%, respectively (Fig. 4C, D), which contrasts with the increase in apoptosis observed when the drugs are administered independent of *p53(RNAi)*. These results suggest that the gold(I) compounds act on the cell cycle, AdT by decreasing the number of mitotic cells and all drugs by decreasing cell death that results in an attenuated *p53(RNAi)* phenotype with increased survival time.

## Discussion

In this study, we used gold(I)-containing small molecules that were designed to improve bioactivity^28^. The ligands used in the complexes may exert biological activity themselves or, by increasing lipophilicity and enhancing cell permeability^28^. In cell culture, the presence of the ligands increases the activity and toxicity of the complexes^28^. Despite the toxicity to normal cells, they demonstrated some selectivity towards cancer cells, which demonstrates that the gold(I) complexes have promising anti-cancer activity. Our results in testing the bioactivity of these compounds in planarians are consistent with observations in cell culture experiments; worms treated with 1 μM of the precursor had a higher survival rate than the ones treated with the same concentration of the complexes. We speculate that this is due to an increased cellular uptake of the complexes, which can be facilitated by the presence of the ligands.

Because cell-cycle irregularities are a hallmark of cancer, reducing cell proliferation or inducing apoptosis have been traditional objectives in anti-cancer drug development^38,39^. The gold(I) complexes used in this study are inhibitors of TR in mammalian cells. Planarians possess TR, GR (Glutathione Reductase) and Thioredoxin Glutathione Reductase (TGR) genes. Of the three enzymes, TGR appears to account for most redox activity^40^. These disulfide reductase enzymes have a cysteine residue in their active site, making them amenable to inhibition by gold(I) complexes^41^. In fact, TGR from parasitic flatworms, such as schistosomes and *Taenia*, can be inhibited by gold-containing compounds^42–44^. As TR has been shown to play an important role in regulating signaling pathways involved cell cycle control^17^, we hypothesized that the gold(I) compounds used in this work would affect mitotic and apoptotic rates in planarians. We found that AdT was the only complex capable of reducing mitotic rates in planarians, suggesting that thiazolidine can either increase the rate of drug uptake or bind to different targets. Thiazolidine is a heterocyclic ring system that has a wide range of biological activity, and when used as a ligand it is capable of improving the anti-cancer activity of small molecules and increasing the anti-proliferative activity^45–47^. Interestingly, all compounds caused an increase in number of apoptotic cells, showing the potential of these drug candidates to promote anti-cancer activity.

Planarians have been used to test pharmacological and carcinogenic compounds and to examine the molecular basis of tumorigenesis^23,48–50^. Using RNAi, we sought to disturb pathways that can cause tumor-like outgrowth formation in planarians and investigated the effect of gold(I) complexes treatment during these genetic perturbations. We chose to examine the *p53* signaling pathways because it is a well-studied tumor suppressor gene in mammals, and knockdown of the planarian homolog results in highly penetrant cell proliferation phenotypes^24^. Gold(I) drug treatment increased the survival of *p53*(*RNAi*) worms, suggesting that the compounds may act on a target or pathway associated with P53 signaling.

One explanation for the increased survival of *p53*(*RNAi*) worms treated with the complexes is that apoptosis was reduced when compared to the vehicle control. Observations from P53-deficient models showing increased apoptosis demonstrate the existence of apoptotic pathways regulated independently of P53^51,52^. One mechanistic hypothesis consistent with our findings is that TR is regulating transcription factors that promote P53-independent apoptosis pathways, and inhibiting TR is disrupting this pathway and decreasing apoptosis. Alternatively, the proposed P53-independent apoptosis pathway induced in *p53* knockdown worms, may involve a protein that is directly targeted by the gold(I) compounds. For example Cisplatin can induce P53-independent apoptosis by increasing expression of NOXA, a pro-apoptotic Bcl-2 family member^53^. Thus, any of the pro-apoptotic Bcl-2 family members like NOXA or PUMA could be responsible for the P53-independent pathway and targeted by the complexes. As planarians possess Bcl-2 family genes and caspase-like genes^32,54^, if these proteins are found to be targeted by the gold(I) complexes - using techniques such as target identification by chromatographic co-elution and drug affinity responsive target stability^55^ - planarians could be used to further examine the effects of gold(I) complexes on these pathways *in vivo*. Discovering the specific molecular targets of the gold(I) complexes in planarians could provide insights into the specific bioactivity of these compounds in a P53-depleted context and further establish planarians as a model for drug discovery purposes.

## Conclusions

Planarians can tolerate treatment with gold(I) complexes, and are absorbed by the worms when added to culture medium. The drugs have an effect on the planarians cell cycle and in *p53(RNAi)* worms they increase survival and decrease apoptosis, resulting in an attenuated *p53(RNAi)* phenotype. Additional studies are needed to determine the exact target of these complexes, so that we can better characterize any P53-independent apoptotic pathways that are inhibited by the complexes and further develop this model for screening new anti-cancer compounds. This study offers an insight into how planarians can be a useful model for drug discovery, providing information on how compounds affect the cell cycle, stem cell population, regeneration and specific pathways that can be disrupted by gene knockdown. RNAi approach can be used in these animals to simulate different disease states and serve as models for toxicity, efficacy and target identification.
